# Sexual Dimorphism in the Expression of Mitochondria-Related Genes in Rat Heart at Different Ages

**DOI:** 10.1371/journal.pone.0117047

**Published:** 2015-01-23

**Authors:** Vikrant Vijay, Tao Han, Carrie L. Moland, Joshua C. Kwekel, James C. Fuscoe, Varsha G. Desai

**Affiliations:** Personalized Medicine Branch, Division of Systems Biology, National Center for Toxicological Research, U.S. Food and Drug Administration, Jefferson, Arkansas, United States of America; CNRS/University Lyon 1, FRANCE

## Abstract

Cardiovascular disease (CVD) is the leading cause of mortality worldwide. Moreover, sex and age are considered major risk factors in the development of CVDs. Mitochondria are vital for normal cardiac function, and regulation of mitochondrial structure and function may impact susceptibility to CVD. To identify potential role of mitochondria in sex-related differences in susceptibility to CVD, we analyzed the basal expression levels of mitochondria-related genes in the hearts of male and female rats. Whole genome expression profiling was performed in the hearts of young (8-week), adult (21-week), and old (78-week) male and female Fischer 344 rats and the expression of 670 unique genes related to various mitochondrial functions was analyzed. A significant (p<0.05) sexual dimorphism in expression levels of 46, 114, and 41 genes was observed in young, adult and old rats, respectively. Gene Ontology analysis revealed the influence of sex on various biological pathways related to cardiac energy metabolism at different ages. The expression of genes involved in fatty acid metabolism was significantly different between the sexes in young and adult rat hearts. Adult male rats also showed higher expression of genes associated with the pyruvate dehydrogenase complex compared to females. In young and adult hearts, sexual dimorphism was not noted in genes encoding oxidative phosphorylation. In old rats, however, a majority of genes involved in oxidative phosphorylation had higher expression in females compared to males. Such basal differences between the sexes in cardiac expression of genes associated with energy metabolism may indicate a likely involvement of mitochondria in susceptibility to CVDs. In addition, female rats showed lower expression levels of apoptotic genes in hearts compared to males at all ages, which may have implications for better preservation of cardiac mass in females than in males.

## Introduction

Cardiovascular disease (CVD) is the leading cause of mortality worldwide [1] and in addition to other risk factors, sex and age also play major role in the susceptibility to CVDs. A number of studies have demonstrated sex-related differences in cardiovascular diseases (CVDs). Systemic hypertension and atrial fibrillation occur at a higher rate in males than females, whereas pulmonary hypertension is more common in females than males [2]. Moreover, women show delayed development of atherosclerosis, lower incidence of heart failure [3–5], and develop heart disease later in life than men [6]. It is thought that steroid sex hormones play a substantial role in sexual dimorphism in CVDs [4, 7, 8]. Also, inherent differences in cardiac morphology and function observed in healthy humans and animal model systems have been proposed as potential risk factors for sex-associated susceptibility to CVDs [9–11]. In females, hearts are smaller than males [12, 13], show greater contractility [11], and better calcium handling [14]. Although there are many studies describing sex-related cardiovascular risk, the molecular basis underlying the differences in the development of CVDs between the sexes is not yet well-defined.

Another potential determinant in sex-related differences in CVDs is mitochondria. These organelles provide more than 90% of the energy essential for cardiac tissue to perform physiological and biochemical functions. Paradoxically, mitochondria are also major sites for generation of oxygen free radicals, which perform a central role in the pathogenesis of CVDs [15]. Even under normal physiological conditions, mitochondria are a prime source of reactive oxygen species. Complex I and complex III of the electron transport chain are believed to be the major sites for reactive oxygen species production [16]. It has recently been demonstrated that the ratio between electrons entering the respiratory chain via FADH2 or NADH determines radical formation; the ratio is low during glucose oxidation whereas fatty acid oxidation increases the ratio [17].

There is considerable evidence indicating an association between defects in mitochondrial function and CVDs. For example, mutations in nuclear and mitochondrial genes encoding mitochondrial proteins associated with oxidative phosphorylation have been shown to cause cardiomyopathy and cardiac defects due to impaired mitochondrial energy production and increased reactive oxygen species production [18–20]. Altered fatty acid oxidation activity within mitochondria has also been linked to cardiac pathology [21]. It has been suggested that mitochondrial dysfunction in cardiomyocytes could lead to decreased energy production, reduced contractility, altered electrical properties and cell death [22]. Another aspect of mitochondria that has been related to heart failure is the dynamic process of mitochondrial fission and fusion. An imbalance between fission and fusion in favor of fission can lead to apoptosis and loss of cardiomyocytes [23]. Altogether, this information suggests a major role of mitochondria in cardiac diseases.

It is therefore, likely that sex-based differences in mitochondrial activity in the heart under normal and pathological conditions could lead to differential outcome in cardiac function between the sexes and thus the susceptibility to CVDs. There are studies that investigated cardiac mitochondria to understand its possible involvement in sex-based differences with CVDs. For example, Colom and colleagues (2007) [12] have demonstrated that cardiac mitochondria are more differentiated with higher phosphorylation capacity in females than in males of 15-month old (adult) Wistar rats. The female Wistar rats also showed lower production of hydrogen peroxide in cardiac mitochondria, further suggesting lower oxidative damage in female rats compared to males. Higher activities of mitochondrial complexes III-V in heart have also been shown in female monkeys compared to males [24]. Altogether, this implies that inherent variations in cardiac mitochondrial activity may exist between the sexes, which can contribute to sex-related differences in CVDs. Sexual dimorphism in mitochondrial activity is further underscored by the fact that estrogen regulates mitochondrial biogenesis, oxygen consumption, and energy production [25]. Interestingly, it has been suggested that the genes on the X chromosome can affect the development of CVDs through alteration of mitochondrial function [26].

As stated earlier, mitochondria are a prime source of reactive oxygen species. These reactive species are believed to be responsible for the aging process, which in turn, is one of the major risk factors in the development of CVDs [27, 28]. Thus far, a number of studies have been conducted in laboratory animals to independently examine the influence of sex or age on cardiac mitochondria. These studies have reported sex-based differences in calcium uptake in cardiac mitochondria of adult rats [29] and in H_2_O_2_ production and oxidative damage in cardiac muscle mitochondria in 15-months old (adult) rats [12]. Thus, these studies were limited to evaluation of sex differences at a particular age. On the other hand, influence of age was assessed in cardiac mitochondrial gene expression in 6- and 22-months old rats [30] and mitochondrial enzyme activities and gene expression in heart and liver of neonate, 3- and 18-months old rats [31]. These studies were geared towards influence of aging on mitochondria, but not sex differences. Therefore, information related to differences in cardiac mitochondrial activity between the sexes at different life stages is sparse. In addition, studies conducted thus far have evaluated only certain aspects of mitochondrial function, such as oxidative capacity or activities of oxidative phosphorylation complexes, whereas mitochondrial function involves the complex interplay of approximately 1500 genes [32]. Altogether, knowledge provided by these investigations is inadequate to understand the precise mechanism underlying the relationship between mitochondria and sex-related differences in heart disease. To address this knowledge gap, transcriptional profiling of 670 mitochondria-related genes was performed in the hearts of male and female Fischer 344 rats at three different ages (young (8-week), adult (21-week), and old (78-week)) to identify basal differences in cardiac mitochondria between the sexes.

Considering a key role of mitochondria in heart function and pathology we hypothesized that sexual dimorphism in mitochondrial activity in hearts may significantly influence the differential susceptibility to CVDs between the sexes. Therefore, we primarily focused on energy pathways in rat heart. The results demonstrated a significant disparity in expression of genes involved in FA metabolism, pyruvate dehydrogenase (PDH) complex, oxidative phosphorylation, and apoptosis between the sexes at different ages. These findings provide insights into the mechanisms of mitochondrial involvement in sex-based differences in heart diseases and also may aid in designing novel therapeutic strategies or interventions to limit cardiac pathologies.

## Materials and Methods

### Animal husbandry and study design

Male and female Fischer 344 (F344) rats obtained from the National Center for Toxicological Research (NCTR) breeding colony were fed NIH-31 diet and water *ad libitum* and housed under AAALAC (Association for Assessment and Accreditation of Laboratory Animal Care) approved conditions of 12/12-hr light/dark cycle, and were maintained at 23°C with a relative humidity of 50%. The studies were performed with the approval of the NCTR’s Institutional Animal Care and Use Committee. A large rat life-cycle study was conducted in our laboratory to measure transcriptional levels of genes in various tissues of male and female untreated control F344 rats at up to 9 different ages (2, 5, 6, 8, 15, 21, 52, 78, and 104 weeks) to determine sexually dimorphic gene expression during aging [33–35]. Median lifespan of F344 male rats is about 31 months (135 weeks) and 29 months (126 weeks) in females [36]. At each age, animals were humanely euthanized by CO_2_ asphyxiation and various organs were removed quickly, flash frozen in liquid nitrogen, and stored at -80°C until further investigation. The present study was part of a project funded by the Office of Women’s Health, U.S. Food and Drug Administration. This project was primarily designed to determine sex-related differences in basal transcriptional levels of genes in hearts of male and female untreated F344 rats at three different ages to get important insights into differential susceptibility of heart to drug toxicity and pathology between the sexes. For the present study, hearts from 5 male and 5 female rats at three different ages (young (8-week), adult (21-week), and old (78-week)) were used for investigation of sex-related differences in expression levels of genes. No decline in body weights of male and female rats was observed earlier than 78 week of age [34]. Therefore, 78-week aged rats were chosen to represent old aged group. The manuscript describes changes in expression of only mitochondria-related genes in hearts of these animals.

In microarray experiment, proper randomization of samples is critical to control the influence of systematic noise that can be introduced into the data at different steps during measurement of gene expression. Therefore, the experiment was designed in such a way that RNA samples from males and females of same age were kept in the same batch to accurately measure sex differences in gene expression levels without any batch effect as confounding factor. Since animals of different ages were in different batches (thus batch effect would confound the age effect), neither expression change with age nor sex*age interaction was evaluated for this project. Analysis of data to determine age effect or sex*age interaction might result in false-positive outcome for the randomization design that was used. The present study, therefore, discussed only sex-related differences in expression levels of mitochondria-related genes in rat heart at 3 different ages.

### Isolation of RNA from heart tissues

The frozen hearts were individually ground into powder in liquid nitrogen using a mortar and pestle chilled on dry ice. Total RNAs were extracted from heart tissue powder using the Qiagen RNeasy mini kit (Qiagen Inc., Valencia, CA) with some modifications. In brief, heart tissue powder was homogenized using the FastPrep homogenizer (Qbiogene, Inc., Carlsbad, CA) for 2 x 40 seconds in RLT buffer (Qiagen, Inc.). The homogenate was centrifuged at 4000 x g for 5 min. To the resultant supernatant, an equal volume of ethanol was added followed by vigorous shaking. The samples were immediately applied to an RNeasy mini column (Qiagen, Inc.) and centrifuged at 4000 x g for 10 min. This was followed by on-column treatment with DNase I to remove any residual DNA from the RNA samples before final elution. The purity and yield of each RNA sample were determined using the NanoDrop ND-1000 Spectrophotometer (Thermo Scientific, Wilmington, DE). The ratios of A260/280 from all the RNA samples ranged from 1.8 to 2.0. The quality of the extracted RNAs was evaluated using the Agilent 2100 Bioanalyzer (Agilent Technologies, Inc., Santa Clara, CA). RNA samples with RNA Integrity Numbers (RINs) of 8.0 or above were used for gene expression measurements.

### Gene expression measurements

Gene expression was measured using Agilent Whole Rat Genome Microarrays (4 x 44k format; Agilent Technologies, Inc.). Total RNAs (500ng) were labeled with cyanine dye (Cy3-CTP) using the Low RNA Input Linear Amplification Kit according to the manufacturer’s protocol (Agilent Technologies, Inc.). For each reaction, cRNA yields and specific activities were determined using the NanoDrop ND-1000 Spectrophotometer. Only the cRNAs with yields >1.65 µg and specific activities > 9.0 pmol of dye per µg cRNA were used for hybridization. Labeled cRNAs were hybridized to microarray slides (4 arrays per slide) following the Agilent One-Color Microarray-Based Gene Expression Analysis Protocol (V5.5, Agilent Technologies, Inc.). The hybridized slides were scanned using a GenePix 4000B scanner (Axon Instruments, Sunnyvale, CA) at 5µm resolution using appropriate photomultiplier tube gain settings to obtain the highest intensity with <1% saturated pixels. The resulting images were analyzed by quantifying the Cy3 fluorescence intensity at each of the 45,018 gene spots (features) on each array using the Agilent Feature Extraction Software (V9.5). The median fluorescence intensity of all the pixels within each feature was taken as the intensity value for that feature.

### Data analysis

A total of 30 raw data files for 30 arrays (samples) (5 arrays for each sex at 3 ages) were obtained from the Agilent Feature Extraction Software (Agilent Technologies, Inc.). These raw data files were then uploaded to ArrayTrack, an in-house microarray data management and analysis software system [37]. The raw intensity data for all 30 samples were exported from ArrayTrack as a single Excel spreadsheet. This Excel spreadsheet was used as an input file for further data preprocessing, normalization, differential gene expression and GO (Gene Ontology) analysis using SAS 9.1.3 (SAS institute Inc., Cary, NC) as described below. All the 30 raw microarray data files and preprocessed normalized data are accessible at NCBI’s Gene Expression Omnibus (GEO) website and the GEO accession number is GSE58204 (http://www.ncbi.nlm.nih.gov/geo/query/acc.cgi?acc=GSE58204).

There were 45,018 features (probes) on each array with gene annotations available for 28,552 probes (18,157 unique genes with variable number of probes per gene) [33]. Average probe intensity was calculated for each gene, then non-expressed genes were removed and data was normalized using 75^th^ percentile scaling. Analysis of Variance (ANOVA) using generalized linear model procedure (proc glm) in SAS was performed to measure statistically significant difference in gene expression between females and males (p<0.05) at each of 3 age groups. Of the 18,157 genes on the arrays, 670 unique nuclear genes were determined to be related to mitochondrial function (S1 Table). Fold changes (female vs. male) and p-values for these 670 genes were extracted and GO analysis was performed to determine whether various pathways represented by expression of these genes were significantly different between females and males at each of the 3 age groups.

### Gene Ontology (GO) analysis

To allow better biological interpretation of the complex gene expression data, the genes were further classified into different GO terms based on biological processes or molecular functions associated with mitochondria. These GO terms were obtained from the National Center for Biotechnology Information’s (NCBI) FTP website (ftp://ftp.ncbi.nlm.nih.gov/gene/DATA/gene2go.gz). The sex effect on different GO terms was measured by a modified meta-analysis method for combining p-values to interpret the biology [38, 39]. This method has provided important insights into the mechanisms of altered mitochondrial function in our previous studies [40–42].

### Verification of differentially expressed genes by quantitative real time PCR (qRT-PCR)

Five genes selected for verification by qRT-PCR of their relative expression levels were significantly different between the sexes as estimated by microarray analysis. These included, acetyl-Coenzyme A acyltransferase 2 (mitochondrial 3-oxoacyl-Coenzyme A thiolase) (Acaa2) involved in FA metabolism, pyruvate dehydrogenase kinase, isoenzyme 4 (Pdk4) involved in regulation of glucose metabolism, bcl2-associated death promoter (Bad) involved in apoptosis, NADH dehydrogenase (ubiquinone) 1 alpha subcomplex 1 (Ndufa1) associated with complex I of the electron transport chain, and cytochrome c oxidase subunit VIIb (Cox7b) associated with complex IV of the electron transport chain. These differentially expressed genes also represent GO terms significantly different between the sexes. The transcription level of β-actin (Actb) was unaltered by sex or age and therefore was used as an endogenous control gene.

RNA samples used for real-time PCR were from the same aliquots of RNA used for the microarray experiment. For each gene, the relative mRNA level was measured using the Taqman gene expression assay kit consisting of a set of sequence-specific primers and a Taqman probe with a fluorescent reporter dye FAM and a non-fluorescent quencher moiety attached to the 5’ and 3’ ends, respectively (Applied Biosystems, Foster City, CA). Gene expression assays for Acaa2 (Rn00590503_ml), Pdk4 (Rn00585577_ml), Bad (Rn00575519_ml), Ndufa1 (Rn01457343_gl), Cox7b (Rn00822088_gl), and Actb (Rn00667869_ml) were purchased as Assays-on-Demand kits (Applied Biosystems). One µg of total RNA was reverse transcribed using a High Capacity RNA-to-cDNA kit according to the manufacturer’s protocol (Applied Biosystems). Twenty nanograms of the resultant cDNA was then used as the template in a 20 µl Taqman real time PCR reaction on an ABI 7900HT Fast Real-Time PCR system using the Taqman Universal Master Mix II with UNG kit. Each sample was run in triplicate and the mean C_t_value was used to calculate the relative fold change in female rats compared to male rats using the 2^-ΔΔCt^ method [43].

## Results

Transcriptional levels of 670 unique mitochondria-related genes were evaluated in the hearts of young (8-week), adult (21-week), and old (78-week) male and female F344 rats. The list of 670 genes was created using the previously established list of 542 mitochondria-related genes [40] plus 128 genes with mitochondria-associated annotation from NCBI’s database (S1 Table). A significant (p<0.05) sexual dimorphism in expression levels of 46, 114, and 41 genes was found in young, adult and old rats, respectively (S2 Table). Only one gene, *Mthfd2* (methylenetetrahydrofolate dehydrogenase (NADP+ dependent) 2, methenyltetrahydrofolate cyclohydrolase) was common among aforementioned significant sexual dimorphic genes, which demonstrated consistently higher expression in males than in females at all ages. Altogether, 68 mitochondrial-related GO terms were established and the differential expression of 670 genes as a group in various pathways was evaluated using GO analysis. Differences in expression levels of a majority of genes between the sexes were subtle. Previous studies in our laboratory have demonstrated that the fold changes of genes related to mitochondria structure and function are often less than 1.5 [40–42]. Application of multiple testing adjustments to p-values may result in loss of significant mitochondria-related genes with subtle changes in the expression. Therefore, only p-value (<0.05) cutoff without any fold change cutoff was applied followed by gene ontology analysis to understand the biology. Also, although changes in expression levels are subtle, the GO term (molecular function/biological processes) may show a significant effect because data analysis takes into account the collective effects (correlation) of all the genes associated with that GO term.

Analysis of difference in gene expression together demonstrated a significant sex effect on 25 GO terms (biological process/molecular function) at various ages. All evaluated GO terms with the number of genes in each GO term and p-values for all 3 ages are presented in S3 Table. There are GO terms that may have gene(s) common among them because the proteins encoded by these genes perform important function in different pathways and therefore are categorized under functionally relevant various GO terms. The major difference in the number of unique genes (670) presented in S1 Table vs total number of genes (771) per GO terms in S3 Table is because of a category named “oxidative phosphorylation”, which includes 85 genes already present in GO categories of complex I-V. This was performed to determine the sex-based differences on overall oxidative phosphorylation.

The primary objective of the present study was to evaluate the expression levels of genes associated with energy metabolism in hearts of male and female F344 rats for better understanding of a likely role of mitochondria in differential susceptibility to cardiovascular diseases between the sexes. Therefore, the manuscript discusses only three key GO terms involved in energy metabolism (oxidative phosphorylation, fatty acid metabolism, pyruvate dehydrogenase complex) and also a GO term related to apoptosis considering implications of apoptotic process in the development of cardiovascular diseases. These GO terms showed a significant sex effect (Table 1) and are discussed below.

**Table 1:** Sexually dimorphic gene expression in Fischer 344 rats.

**Gene Description (Gene Symbol)**	**GenBank Acc ID**	**8 weeks**		**21 weeks**		**78 weeks**	
		**p**	**FC**	**p**	**FC**	**p**	**FC**
*Oxidative Phosphorylation* (**85**) ^a^		0.971		0.109		0.034 ^b^	
*Complex I (NADH—Ubiquinone dehydrogenase)* (**36**) ^a^		0.990		0.105		0.039 ^b^	
NADH dehydrogenase 1 alpha subcomplex, 1 (Ndufa1)	XM_343760	0.690	1.022	0.346	0.855	0.017 ^c^	1.144
NADH dehydrogenase 1 alpha subcomplex, 2 (Ndufa2)	XM_214570	0.645	1.023	0.071	0.755	0.041 ^c^	1.075
NADH dehydrogenase complex I, assembly factor 1 (Ndufaf1)	XM_215814	0.856	0.992	0.031 ^c^	0.835	0.907	1.009
NADH dehydrogenase 1 beta subcomplex, 2 (Ndufb2)	XM_342664	0.437	1.054	0.264	0.846	0.045 ^c^	1.126
NADH dehydrogenase Fe-S protein 6 (Ndufs6)	NM_019223	0.614	1.028	0.174	0.806	0.037 ^c^	1.119
NADH dehydrogenase flavoprotein 1 (Ndufv1)	NM_001006972	0.851	1.011	0.022 ^c^	0.831	0.527	1.109
NADH dehydrogenase flavoprotein 3 (Ndufv3)	NM_022607	0.516	1.031	0.103	0.754	0.016 ^c^	1.115
*Complex IV (Cytochrome c oxidase)* (**20**) ^a^		0.910		0.104		0.009 ^b^	
cytochrome c oxidase assembly protein 11 (Cox11)	BF567145	0.932	0.987	0.008 ^c^	2.019	0.312	1.233
cytochrome c oxidase subunit VIIa polypeptide 2 (Cox7a2)	NM_022503	0.421	1.059	0.365	0.885	0.036 ^c^	1.174
cytochrome c oxidase subunit VIIb (Cox7b)	NM_182819	0.248	1.056	0.163	0.801	0.013 ^c^	1.213
cytochrome c oxidase subunit VIIIa (Cox8a)	AI102505	0.718	1.148	0.532	1.343	0.009 ^c^	1.673
surfeit 1 (Surf1)	NM_172068	0.648	1.016	0.228	0.863	0.017 ^c^	1.124
*FA Metabolism* (**53**) ^a^		0.007 ^b^		0.001 ^b^		0.950	
acetyl-CoA acyltransferase 2 (Acaa2)	NM_130433	0.033 ^c^	1.103	0.002 ^c^	0.739	0.580	1.061
acyl-CoA dehydrogenase, C-4 to C-12 straight chain (Acadm)	NM_016986	0.051	1.085	0.002 ^c^	0.899	0.479	1.065
acyl-CoA dehydrogenase, C-2 to C-3 short chain (Acads)	BM986570	0.022 ^c^	1.286	0.110	0.744	0.937	1.011
acetyl-CoA acetyltransferase 1 (Acat1)	NM_017075	0.779	0.990	0.050 ^c^	0.887	0.778	1.016
acyl-CoA thioesterase 2 (Acot2)	NM_138907	0.152	1.137	0.011 ^c^	1.240	0.267	1.181
acyl-CoA synthetase long-chain family member 3 (Acsl3)	NM_057107	0.654	0.982	0.004 ^c^	1.335	0.443	0.944
acyl-CoA synthetase long-chain family member 4 (Acsl4)	NM_053623	0.010 ^c^	0.850	0.261	1.362	0.010 ^c^	0.795
acyl-CoA synthetase long-chain family member 6 (Acsl6)	NM_130739	0.056	0.856	0.008 ^c^	0.790	0.299	1.142
cytochrome P450, family 11, subfamily a, polypeptide 1 (Cyp11a1)	NM_017286	0.804	1.044	0.008 ^c^	0.575	0.575	1.143
enoyl CoA hydratase 1, peroxisomal (Ech1)	NM_022594	0.017 ^c^	1.176	0.476	0.931	0.344	1.114
hydroxyacyl-CoA dehydrogenase/3-ketoacyl-CoA thiolase/enoyl-CoA hydratase (trifunctional protein), beta subunit (Hadhb)	NM_133618	0.036 ^c^	1.115	0.382	0.943	0.200	1.237
3-hydroxy-3-methylglutaryl-CoA synthase 2 (mitochondrial) (Hmgcs2)	AI232320	0.023 ^c^	1.618	0.824	0.929	0.550	1.271
malonyl-CoA decarboxylase (Mlycd)	AI232703	0.033 ^c^	1.180	0.784	0.958	0.086	1.225
3-oxoacyl-ACP synthase, mitochondrial (Oxsm)	XM_001068016	0.493	0.949	0.013 ^c^	0.818	0.785	1.026
propionyl CoA carboxylase, alpha polypeptide (Pcca)	XM_001075496	0.032 ^c^	1.058	0.059	0.902	0.377	1.065
*Pyruvate dehydrogenase complex* (**11**) ^a^		0.175		0.002 ^b^		0.068	
dihydrolipoamide S-acetyltransferase (Dlat)	NM_031025	0.146	0.900	0.041 ^c^	0.898	0.438	1.051
pyruvate dehydrogenase complex, component X (Pdhx)	XM_230327	0.225	0.895	0.040 ^c^	0.794	0.994	0.999
pyruvate dehydrogenase kinase, isozyme 1 (Pdk1)	NM_053826	0.085	1.150	0.009 ^c^	0.689	0.091	1.169
pyruvate dehydrogenase kinase, isozyme 4 (Pdk4)	AI031053	0.002 ^c^	1.443	0.018 ^c^	1.762	0.355	1.248
pyruvate dehyrogenase phosphatase catalytic subunit 1 (Pdp1)	NM_019372	0.724	1.014	0.610	0.980	0.043 ^c^	1.267
*Apoptosis* (**65**) ^a^		0.005 ^b^		0.003 ^b^		0.008 ^b^	
apoptosis, caspase activation inhibitor (Aven)	XM_230438	0.381	1.063	0.038 ^c^	0.858	0.570	1.072
BCL2-associated agonist of cell death (Bad)	NM_022698	0.695	0.977	0.040 ^c^	0.736	0.943	0.993
Bcl2-associated X protein (Bax)	XM_001061020	0.020 ^c^	1.055	0.425	0.930	0.941	0.994
BCL2/adenovirus E1B interacting protein 2 (Bnip2)	XM_217191	0.254	0.935	0.012 ^c^	0.861	0.608	1.106
BCL2/adenovirus E1B interacting protein 3-like (Bnip3l)	AI175871	0.805	0.952	0.059	0.585	0.00004 ^c^	0.471
caspase 1 (Casp1)	NM_012762	0.083	0.928	0.005 ^c^	0.726	0.175	0.841
caspase 12 (Casp12)	NM_130422	0.140	0.891	0.002 ^c^	0.792	0.071	0.779
caspase 2 (Casp2)	NM_022522	0.315	0.985	0.040 ^c^	1.294	0.651	0.987
caspase 3 (Casp3)	AI059604	0.329	0.865	0.004 ^c^	0.564	0.201	0.673
caspase 4, apoptosis-related cysteine peptidase (Casp4)	NM_053736	0.0004 ^c^	0.852	0.016 ^c^	0.698	0.160	0.837
caspase 7 (Casp7)	NM_022260	0.068	0.961	0.597	0.954	0.010 ^c^	1.147
death-associated protein (Dap)	NM_022526	0.280	0.924	0.060	0.813	0.040 ^c^	0.856
death associated protein kinase 1 (Dapk1)	XM_225138	0.894	1.005	0.442	0.906	0.032 ^c^	0.810
death associated protein kinase 2 (Dapk2)	XM_578739	0.155	0.856	0.001 ^c^	0.682	0.328	0.862
death associated protein kinase 3 (Dapk3)	NM_022546	0.973	1.002	0.049 ^c^	0.813	0.368	0.929
death associated protein-like 1 (Dapl1)	XM_342436	0.029 ^c^	0.529	0.848	0.906	0.938	1.031
DNA fragmentation factor, alpha subunit (Dffa)	NM_053679	0.072	0.882	0.009 ^c^	0.785	0.481	0.949
DNA fragmentation factor, beta subunit (Dffb)	NM_053362	0.921	1.025	0.038 ^c^	0.525	0.081	0.648
dihydroorotate dehydrogenase (quinone) (Dhodh)	NM_001008553	0.611	0.958	0.004 ^c^	0.788	0.501	0.953
mitochondrial carrier 2 (Mtch2)	AA892863	0.034 ^c^	1.908	0.539	1.181	0.131	0.782
programmed cell death 10 (Pdcd10)	AA964705	0.491	1.084	0.044 ^c^	0.662	0.384	0.876
programmed cell death 5 (Pdcd5)	XM_214911	0.208	1.052	0.416	0.920	0.011 ^c^	1.161
programmed cell death 6 (Pdcd6)	XM_217732	0.837	0.993	0.018 ^c^	0.722	0.445	1.034
programmed cell death 7 (Pdcd7)	XM_343413	0.051	1.103	0.030 ^c^	1.176	0.725	1.020
TP53 regulated inhibitor of apoptosis 1 (Triap1)	XM_001077518	0.010 ^c^	1.072	0.400	0.916	0.044 ^c^	1.100
voltage-dependent anion channel 1 (Vdac1)	AA875489	0.410	1.085	0.025 ^c^	0.725	0.846	0.982

This table represents a list of sexually dimorphic genes in one or more of the 3 ages (young, adult and old).

^a^ Gene Ontology (GO) term (number of genes evaluated).

^b^ Significant (p< 0.05) sex difference on overall GO term.

^c^ Significant (p< 0.05) sex difference in expression level of each gene.

FC—Fold Change calculated as a ratio of average expression levels in female hearts to male hearts.

p—probability value < 0.05.

### Sex-based differences in young (8-week) rats

**Fatty acid (FA) metabolism**. Out of 53 genes interrogated for this category, 32 genes (60%) had higher expression levels in female rat hearts compared to males, but the effect was significant for only eight genes. Of these eight genes, expression of *Acaa2* (acetyl-CoA acyltransferase 2), *Acads* (acyl-CoA dehydrogenase, C-2 to C-3 short chain), *Ech1* (enoyl CoA hydratase 1, peroxisomal), *Hadhb* (hydroxyacyl-CoA dehydrogenase/3-ketoacyl-CoA thiolase/enoyl-CoA hydratase (trifunctional protein), beta subunit), *Hmgcs2* (3-hydroxy-3-methylglutaryl-CoA synthase 2 (mitochondrial)), *Mlycd* (malonyl-CoA decarboxylase), and *Pcca* (propionyl CoA carboxylase, alpha polypeptide) was higher in females compared to males, and only *Acsl4* (acyl-CoA synthetase long-chain family member 4) had significantly higher expression in males compared to females. Differentially expressed genes had average relative fold-changes ranging from 1.06- to 1.62-fold between the sexes. Altogether, the sex effect on overall FA metabolism was significant (p = 0.007) in young rats. The relative fold change of *Acaa2* between the sexes was confirmed by qRT-PCR (Table 2).

**Table 2:** Verification of significantly altered genes by quantitative real time PCR (qRT-PCR).

**Gene Symbol**	**GenBank Accession ID**	**Microarray (Average fold change ^a^)**	**qRT-PCR** **(Average fold change ^a^ ± SEM)**
		**8-week**	
Pdk4	AI031053	1.44	1.49 ± 0.076
Acaa2	NM_130433	1.10	1.06 ± 0.080
		**21-week**	
Pdk4	AI031053	1.76	2.69 ± 0.590
Acaa2	NM_130433	0.74	0.87 ± 0.060
Bad	NM_022698	0.74	0.89 ± 0.059
		**78-week**	
Ndufa1	XM_343760	1.15	1.41 ± 0.118
Cox7b	NM_182819	1.21	1.64 ± 0.192

^a^ Average fold change in gene expression was calculated as a ratio of average gene expression in female to male heart.

SEM: Standard Error of the Mean (N = 5 rats/group).

**Apoptosis**. A total of 65 genes associated with the apoptotic process (see S3 Table) were evaluated to determine the sex effect. Thirty-seven genes (57%) showed higher expression levels in males compared to females, whereas 28 genes (43%) had higher expression levels in females compared to males. These included both pro- and anti-apoptotic genes. However, only 5 genes exhibited a significant sex difference. Expression of *Casp4* (caspase 4, apoptosis-related cysteine peptidase) and *Dapl1* (death associated protein-like 1) was significantly higher in males compared to females, whereas expression of *Bax* (Bcl2-associated X protein), *Mtch2* (mitochondrial carrier 2), and *Triap1* (TP53 regulated inhibitor of apoptosis 1) was significantly higher in females compared to males. Sex-related differences in the expression levels of these significantly altered genes were between 1.05- and 1.91-fold. The changes in expression levels of 65 genes collectively showed a significant sex effect on apoptosis (p = 0.005) in young rats (Table 1).

### Sex-based differences in adult (21-week) rats

**Fatty acid (FA) metabolism**. GO analysis showed a significant (p = 0.001) sex effect on FA metabolism. Thirty-three of 53 genes (62%) were more highly expressed in male rat hearts compared to females, and the expression of 8 genes was significantly different between the sexes. Expression of *Acaa2* (acetyl-CoA acyltransferase 2), *Acadm* (acyl-CoA dehydrogenase, C-4 to C-12 straight chain), *Acat1* (acetyl-CoA acetyltransferase 1), *Acsl6* (acyl-CoA synthetase long-chain family member 6), *Cyp11a1* (cytochrome P450, family 11, subfamily a, polypeptide 1), and *Oxsm* (3-oxoacyl-ACP synthase, mitochondrial) was significantly higher in male compared to female hearts, whereas expression of *Acot2* (acyl-CoA thioesterase 2) and *Acsl3* (acyl-CoA synthetase long-chain family member 3) was significantly higher in female compared to male hearts. Differences in expression levels of these genes ranged from 1.11 to 1.74-fold between the sexes. The lower expression level of *Acaa2*in male compared to female hearts as measured by microarray analysis was verified by qRT-PCR (Table 2).

**Pyruvate dehydrogenase (PDH) complex**. Out of 11 genes evaluated for the PDH complex, eight genes (73%) had higher expression levels in male hearts compared to females, whereas three genes (27%) had expression levels higher in females compared to males. Of these 11 genes, 4 showed a significant sex effect. Expression levels of *Dlat* (dihydrolipoamide S-acetyltransferase), *Pdhx* (pyruvate dehydrogenase complex, component X), and *Pdk1* (pyruvate dehydrogenase kinase, isozyme 1) was significantly higher in males compared to females, and differences in expression levels between the sexes ranged from 1.11- to 1.45-fold. On the other hand, the expression of *Pdk4* (pyruvate dehydrogenase kinase, isozyme 4), was significantly lower by 1.76 fold in male compared to female hearts. GO ontology analysis showed a significant sex effect (p = 0.002) on the PDH complex in adult rats (Table 1). The increased expression of *Pdk4* in females was confirmed by qRT-PCR (Table 2).

**Apoptosis**. Similar to young rats, a significant sex effect (p = 0.003) was also observed on apoptosis in the hearts of adult F344 rats (Table 1). Fifty-three of 65 genes (82%) evaluated for apoptosis had expression levels higher in male hearts compared to females. Seventeen genes (26%) were significantly different between the sexes; a majority of which were pro-apoptotic genes and had higher expression in males compared to females. These included *Aven* (apoptosis, caspase activation inhibitor), *Bad* (BCL2-associated agonist of cell death), *Bnip2* (BCL2/adenovirus E1B interacting protein 2), *Casp1* (caspase 1), *Casp12* (caspase 12), *Casp3* (Caspase 3), *Casp4* (caspase 4, apoptosis-related cysteine peptidase), *Dapk2* (death-associated kinase 2), *Dapk3* (death-associated protein kinase 3), *Dffa* (DNA fragmentation factor, alpha subunit), *Dffb* (DNA fragmentation factor, 40kDa, beta polypeptide (caspase-activated DNase)), *Dhodh* (dihydroorotate dehydrogenase (quinone)), *Pdcd10* (programmed cell death 10), *Pdcd6* (programmed cell death 6), and *Vdac1* (voltage-dependent anion channel 1). On the other hand, expression levels of pro-apoptotic genes *Casp2* (caspase 2) and *Pdcd7* (programmed cell death 7) were significantly higher in female compared to male hearts. The higher expression level of *Bad* in male compared to female hearts was confirmed by qRT-PCR (Table 2).

### Sex-based differences in old (78-week) rats

**Oxidative phosphorylation**. Energy production within mitochondria is carried out by oxidative phosphorylation, which is composed of five complexes, I through V. The sex effect on this GO term was calculated as a cumulative effect of all five complexes and showed a significant effect on oxidative phosphorylation (p = 0.034). A total of 85 genes were interrogated (see S3 Table), of which the expression of 64 genes (75%) was higher in female compared to male hearts. Ten genes were significantly different between the sexes and had higher expression in females compared to males. These included 5 genes of complex I, one gene of complex III (*uqcrc2*), and 4 genes of complex IV. Among five complexes, a significant sex effect was observed only on complexes I (p = 0.039) and IV (p = 0.009) (see Table 1).

**Complex I (NADH ubiquinone dehydrogenase)**. This GO term included 36 genes (see S3 Table), of which 29 genes (81%) had expression levels higher in female compared to male hearts. A significant sex effect was observed on expression of *Ndufa1* (NADH dehydrogenase (ubiquinone) 1 alpha subcomplex, 1), *Ndufa2* (NADH dehydrogenase (ubiquinone) 1 alpha subcomplex, 2), *Ndufb2* (NADH dehydrogenase (ubiquinone) 1 beta subcomplex, 2), *Ndufs6* (NADH dehydrogenase (ubiquinone) Fe-S protein 6), and*Ndufv3* (NADH dehydrogenase (ubiquinone) flavoprotein 3). Differential expression levels of these genes were subtle and ranged between 1.07- and 1.14-fold. The higher expression level of *Ndufa1* in females was verified by qRT-PCR (Table 2).

**Complex IV (cytochrome c oxidase)**. Fifteen out of 20 genes (75%) evaluated for complex IV (see S3 Table) had higher expression levels in female compared to male hearts. The expression of four genes was significantly different between the sexes. Females showed higher expression of *Cox7a2* (cytochrome c oxidase subunit VIIa polypeptide 2), *Cox7b* (cytochrome c oxidase subunit VIIb), *Cox8a* (cytochrome c oxidase subunit VIIIa), and *Surf1* (surfeit 1) when compared to males. The differential expression levels of these genes ranged from 1.12- to 1.67-fold.. The higher expression level of *Cox7b* was 1.21-fold in female hearts relative to males and was confirmed by qRT-PCR (Table 2).

**Apoptosis**. Forty-three genes (66%) out of 65 evaluated for apoptotic process had higher expression in the hearts of old males compared to old females, while 22 genes (34%) had higher expression in females compared to males. The expression of six genes was significantly different between the sexes and these included *Bnip3l* (BCL2/adenovirus E1B interacting protein 3-like), *Dap* (death-associated protein), and *Dapk1* (death associated protein kinase 1), whose expression was higher in males compared to females, and *Casp7* (caspase 7), *Pdcd5* (programmed cell death 5), and *Triap1* (TP53 regulated inhibitor of apoptosis 1), whose expression was higher in females compared to males. The differential expression levels of these genes ranged from 1.10- to 2.12-fold between the sexes. Altogether, this GO term showed a significant (p = 0.008) sex effect in old rats (Table 1).

## Discussion

The objective of the present study was to obtain insights into the role of mitochondria in sex-related differences and potential susceptibility to CVDs during aging. To accomplish this, expression levels of 670 nuclear genes associated with mitochondrial function were analyzed in young, adult, and old male and female F344 rats to determine sex-related differences, if any, in cardiac mitochondria. A prominent finding of the study was a significant sex-related difference in expression levels of cardiac genes involved in energy metabolism in F344 rats at three different ages.

A large body of evidence indicates association between impaired mitochondrial activity and cardiac pathologies both in humans and rodents. Deficiencies in mitochondrial energy in dilated and hypertrophic cardiomyopathies [44, 45] and defects in genes encoding proteins associated with fatty acid metabolism leading to fatal cardiomyopathy [46] have been reported in humans. Also, a number of transgenic animal models exhibiting mutations in nuclear genes involved in mitochondrial function, including fatty acid metabolism have provided important information on a likely association between mitochondrial dysfunction and progression of the cardiac phenotypes [47–49]. Sex-related differences have been indicated in cardiovascular diseases in humans. Nonetheless, information related to differential mitochondrial activity and development of cardiac diseases between the sexes is lacking in both humans and laboratory animals. Results of the present study therefore, will help fill the knowledge gap concerning contribution of mitochondria to the pathophysiological events in the heart between the sexes.

In heart, fatty acids serve as a prime source of energy. Fatty acids are oxidized via the fatty acid β-oxidation pathway within mitochondria and accounts for 60–90% of cardiac energy [50]. In the present study, a significant sex-related difference was observed in FA metabolism in young (p = 0.007) and adult (p = 0.001) rat hearts (Table 1). In young rats, expression of 32 of 53 genes associated with FA metabolism was higher in female hearts compared to age-matched males, whereas 33 of 53 genes showed higher expression in adult male hearts compared to age-matched females. Interestingly, 18 genes that showed higher expression in female hearts at young age, had lower expression at adult age compared to age-matched male hearts. Only one gene, acyl-CoA acyltransferase (*Acaa2*), also known as 3-ketoacyl-CoA thiolase, was found to be common between differentially expressed genes in young and adult hearts. While *Acaa2*, *Acads*, *Acsl4*, *Ech1*, *Hadhb*, *Hmgcs2*, *Mlycd*, and *Pcca* showed a significant sex effect in young rat hearts, a significant sexual dimorphism was observed in the expression levels of *Acaa2*, *Acadm, Acat1*, *Acot2*, *Acsl3*, *Acsl6*, and *Oxsm* in adult rat hearts. Acyl-CoA synthase long-chain family (Acsl) catalyzes conversion of long-chain fatty acids into fatty-acyl Coenzyme A (CoA) esters, which is a key step prior to the oxidation of long-chain fatty acids within mitochondria. Rats showed differential expression for different members of Acsl family in the heart. Young female rats exhibited significantly lower expression of *Acsl4* whereas adult females had higher *Acsl3* expression and lower *Acsl6* expression compared to age-matched males. Once transported inside the mitochondria, esterified fatty acids undergo oxidation through fatty acid β-oxidation pathway that involves a series of four main enzymes, acyl-CoA dehydrogenase (Acad), enoyl-CoA hydratase (Ech), hydroxyacyl-CoA dehydrogenase (Hadh), and ketoacyl-CoA thiolase, resulting in acetyl-CoA molecules as the final product. In young female hearts, acyl-CoA dehydrogenase for short chain fatty acids (*Acads*), Ech1, *Hadha*, and *Acaa2*, associated with each of the four steps involved in β-oxidation of fatty acid, respectively, had expression levels higher compared to young males. In contrast to the higher level of expression of Acads in young female hearts, the expression of the gene for the medium chain-specific form of this enzyme (Acadm) was higher in the hearts of adult males than females. Unlike in young female hearts, expression of *Acaa2*, which catalyzes the final step of the β-oxidation pathway, was lower in adult female hearts compared to adult males. There is not enough information related to sex differences in FA metabolism in heart. Results of the present study provide important insights into the sex differences in cardiac FA metabolism in rats. Interestingly, expression of acetyl-CoA acetyltransferase 1 (*Acat1*), which is involved in ketone breakdown during the processing of fats, was higher in adult male hearts compared to age-matched females.

Glucose, lactate, amino acids, and ketone bodies can also be utilized as alternative metabolic fuels by the heart under certain conditions [51]. Glucose oxidation via glycolysis accounts for 10–40% of total cardiac energy [52]. The pyruvate produced during glycolysis is transported across the outer mitochondrial membrane for further oxidation to acetyl-CoA by the PDH complex located within the inner mitochondrial membrane. Dephosphorylation of PDH by PDH phosphatase activates the enzyme whereas phosphorylation by PDH kinase (PDK) inactivates the enzyme [51]. In the present study, a significant sex effect (p = 0.002) was observed on the expression of the PDH complex only in adult hearts. Eight out of 11 genes (73%) evaluated for the PDH complex had higher expression levels in adult male hearts compared to females. Among four genes that showed a significant sex effect, the expression of only pyruvate dehydrogenase kinase, isoform 4 (*Pdk4*) was significantly lower in male hearts compared to females. Pdk4 is the dominant isoform in the heart [53]. Higher levels of this isoform have been related to lower PDH activity and inhibition of the glucose pathway in cardiomyocytes during hibernation in mammals [54]. In view of this, significantly lower expression of *Pdk4* in adult male hearts compared to females may suggest higher PDH activity and increased glucose pathway in adult male hearts. As mentioned earlier, adult hearts also showed a significant sex effect on genes associated with FA metabolism; males showing higher expression compared to age-matched females. These findings thus indicate that both fatty acids and glucose may be utilized as sources of energy for adult male rat hearts, possibly to meet greater energy demands compared to adult females. In view of this, one may assume a greater risk for development of cardiac dysfunction or pathologies in adult male rats than in females, if fatty acid metabolism and/or pyruvate dehydrogenase complex is impaired. In the healthy human heart, in addition to fatty acids as a source of fuel, energy is derived from glucose oxidation in mitochondria [55]. It has been suggested that many of the metabolic events for selection of fuel and capacity for ATP generation in the heart are regulated at the transcriptional level [56].

Acetyl-CoA derived from fatty acids and pyruvate enters the tricarboxylic acid cycle (TCA) for further oxidation. Both the fatty acid β-oxidation pathway and the TCA cycle provide FADH_2_ and NADH substrates essential for ATP generation by oxidative phosphorylation. Oxidative phosphorylation is a complex system comprised of a respiratory chain of multi-subunit protein complexes I through IV, coupled with phosphorylation of ADP to ATP by ATP synthase (complex V). To date, only a few animal studies have evaluated sex-related differences in specific aspects of mitochondrial energetics in the heart at different ages [12, 24, 29, 57]. Yan et al (2004)[24] showed a significantly higher expression of proteins associated with complexes III, IV, and V of oxidative phosphorylation in the hearts of old female monkeys compared to age-matched males and that this sex difference was absent in the hearts of young monkeys. Also, the rate of cardiac mitochondrial oxygen consumption was higher in old female monkeys compared to age-matched males [24]. A significant gender difference in cardiac mitochondria has also been reported in 15-month old (adult) Wistar rats that showed a greater mitochondrial oxidative potential in hearts of females compared to males [12]. Corroborating these reports is the significant sexual dimorphism in the expression of genes associated with oxidative phosphorylation observed only in old rat hearts in the present study. Sixty-four of 85 genes (75%) associated with oxidative phosphorylation had higher expression levels in female hearts compared to age-matched male hearts. However, only complex I (p = 0.038) and IV (p = 0.009) were significantly different between the sexes. In both complexes, expression levels of a majority of genes, 29 of 36 genes (81%) in complex I and 15 of 20 genes (75%) in complex IV, were higher in females compared to males. Although, complexes II, III, and V did not show a significant sex effect, it should be noted that more than 50% of the 29 genes evaluated for these three complexes had higher expression levels in female hearts compared to males in old rats.

In the present study, higher expression levels of genes associated with oxidative phosphorylation in old female hearts compared to males may suggest a greater oxidative potential of cardiac mitochondria in females compared to males. Such inherent differences in the transcriptional levels of genes associated with energy production between the sexes may significantly contribute to differences in cardiac function and thus the susceptibility to cardiac diseases between the sexes. Studies have demonstrated a greater cardiac contractility in women than in men and better preservation of myocardial mass in women than men during aging [11, 58, 59]. This could, in part, be related to differential cardiac expression of genes involved in mitochondrial energy metabolism between the sexes. Also, sex-based difference in left ventricular function has been reported in old (25 month-old) F344 rats where males demonstrated impaired systolic performance compared to age-matched females, and this was attributed to intrinsic differences in cardiac physiology [9].

Moreover, sex-related differences in cardiac disease might be related to differences in the mechanism responsible for protection of the myocardial structure between the sexes. Loss of cardiomyocytes, which can occur as a result of apoptosis, has been indicated as an important feature in the development of cardiac failure [60] and aging of the heart [61, 62]. Sex difference in loss of cardiomyocytes has been described in aging human heart that showed a greater decline in number of ventricular myocytes in males than in females [59]. Although, histological examination of heart tissues was not performed in F344 rats in the present study, significant sex-related differences observed in expression levels of genes involved in apoptosis in hearts of these rats suggest a greater myocardial loss in male hearts than in female hearts. At all three ages, a majority of pro-apoptotic genes (*Bad*, *Bnip3l*, *Casp3*, *Dap*, *Dapk1*, *Dapk2*, *Dapk3*, *Dffa*, *Dffb*, *Pdcd10*, *Pdcd6*, *Vdac1*) showed higher expression in male hearts compared to females. It has been suggested that apoptosis of cardiomyocytes can lead to reduced contractile force and thinning of myocardial wall [63], which could impair performance of cardiac function. Such differential expression of apoptotic genes in hearts between the sexes may be responsible for better maintenance of myocardial mass and function, leading to possibly lesser prevalence of heart diseases, and longer life-span in females than males in animals and humans [4, 9, 64].

Protection of females against cardiovascular risks has been largely attributed to estrogen [3, 7, 65]. Anti-apoptotic action of estrogen has been proposed as a potential mechanism [66, 67]. In view of this, one may assume increased apoptosis in aging female hearts due to decline in estrogen. In the present study, however, female F344 rats showed lower expression of genes involved in apoptosis compared to age-matched males at all ages. Olivetti and colleagues (1995)[59] have reported that loss of cardiomyocytes in both ventricles in women is insignificant compared to men during aging, further suggesting that other factors besides sex hormones may influence cell death of cardiomyocytes with age.

In summary, the present study showed sexual dimorphism in the transcriptional levels of genes associated with FA metabolism, PDH complex, and oxidative phosphorylation in hearts of F344 rats. It appears that energy requirement in young female hearts might be higher in comparison to young males and is met by increased transcription of genes associated with FA metabolism. On the other hand, both fatty acids and glucose might be utilized as sources of energy to satisfy a greater energy demand in adult male hearts compared to females. However, old female rats seem to exhibit better cardiac potential for ATP production as evidenced by significantly higher expression of a majority of genes associated with oxidative phosphorylation compared to old males. Differential selection of energy sources in F344 rat hearts between sexes at various life stages may have implications for sex differences in cardiac function. Moreover, changes in expression of pro-apoptotic genes observed in F344 rat hearts support the concept that differential apoptosis may be responsible for better preservation of cardiac mass in females than in males. This again, may contribute to sex-related differences in susceptibilities to CVDs.

The molecular mechanisms underlying sex-based differences in development of CVDs are multi-factorial. The basal differences in transcriptional levels of cardiac energy mechanics between the sexes observed in F344 rats in the present study may, in part, explain a possible involvement of mitochondria in sex-related differences in the prevalence and pathologies of various CVDs. However, the present analyses were exploratory and the findings may not be conclusive at this stage. Replication experiments could be conducted to gather more evidence for the association between sex and mitochondrial-related gene expressions. The expression data of mitochondria-related genes in the present study are from healthy untreated rat hearts and may serve as a reference in future studies designed to verify mitochondrial role in differences in CVDs between the sexes.

## Supporting Information

S1 Table List of 670 mitochondria-related genes. (XLSX)

S2 Table Sexually dimorphic genes in hearts of Fischer 344 rats. (XLSX)

S3 Table List of GO terms evaluated using rat heart gene expression data. (XLSX)
